# Current human brain applications and challenges of dynamic hyperpolarized carbon-13 labeled pyruvate MR metabolic imaging

**DOI:** 10.1007/s00259-021-05508-8

**Published:** 2021-08-25

**Authors:** Yan Li, Daniel B. Vigneron, Duan Xu

**Affiliations:** 1 Department of Radiology and Biomedical Imaging, UCSF Radiology, University of California, 185 Berry Street, Ste 350, Box 0946, San Francisco, CA 94107, USA

**Keywords:** Hyperpolarized, Metabolic imaging, Carbon-13, Lactate, Pyruvate

## Abstract

The ability of hyperpolarized carbon-13 MR metabolic imaging to acquire dynamic metabolic information in real time is crucial to gain mechanistic insights into metabolic pathways, which are complementary to anatomic and other functional imaging methods. This review presents the advantages of this emerging functional imaging technology, describes considerations in clinical translations, and summarizes current human brain applications. Despite rapid development in methodologies, significant technological and physiological related challenges continue to impede broader clinical translation.

## Introduction

Metabolism is key to the comprehensive assessment of tissue conditions and functions. Alterations in metabolites often precede anatomic and microstructural changes. Improved characterization of metabolism will facilitate the understanding of disease mechanisms, enable early detection and intervention, as well as identify new avenues for the development of therapeutic strategies. Emerging personalized and precision medicine approaches have highlighted the need of advanced neuroimaging methods to detect metabolism noninvasively. However, measuring metabolism in vivo faces multiple challenges, including low metabolite concentrations, rapidly varying metabolic conditions, and slow signal detection rate. Over the years, numerous techniques have been used to interrogate the metabolic pathways and provided valuable insights into metabolic biomarkers. This review will concentrate on a novel real-time dissolution dynamic nuclear polarization (d-DNP) hyperpolarized (HP) carbon-13 (^13^C)–labeled pyruvate MR metabolic imaging with its advantages, considerations in clinical translation, current human brain data, and then its challenges.

## Advantages of dynamic hyperpolarized ^13^C MR metabolic imaging

Traditionally, MRI has offered the ability to detect steady-state metabolites in tissue using spectroscopic methods. Major metabolites that are routinely quantified in the in vivo brain using proton (^1^H) MR spectroscopy (MRS) are choline, creatine, N-acetyl aspartate (NAA), lactate (detectable under certain conditions), and other less concentrated or J-coupled metabolites such as glutamate and gamma-aminobutyric acid (GABA). These metabolites are crucial indicators of tissue function. For example, as a membrane-bound compound, high choline results from faster membrane turnover (more free, detectable choline), indicating either rapid brain maturation in newborns [1, 2] or degeneration such as in cases of brain tumors [3, 4]; NAA is a neuronal marker, with low levels indicating loss of neurons or neuronal function; glutamate and GABA, the main excitatory and inhibitory neurotransmitters in the brain, have been correlated to the symptom severity in neurologic and psychiatric diseases [5–7]. While these metabolites can indicate brain disorders, they are generally nonspecific and must be used in conjunction with imaging or other clinical indicators for diagnostic purposes. The recent detection of 2-hydroxyglutarate (2HG) using ^1^H MRS [8–10], a hallmark of isocitrate dehydrogenase (IDH) mutation in gliomas [11, 12], has opened new avenues of exploring cancer-specific metabolic pathways noninvasively. While it provides estimates of steady-state levels of metabolites associated with biological properties of the tumor, they are not able to assess rapid biologic changes within the tumor.

PET is another imaging modality that provides molecular and functional information using radioactive isotopes that have advanced the diagnosis and detection of pathologies. The most common radionuclides include fluoride-18 (^18^F) and carbon-11 (^11^C). The half-life or decay rate, 110 min and 20 min for ^18^F and ^11^C, determines when cyclotron radio-pharmacy has to produce the imaging agents and the time for injecting the probes. The recent development of PET/MR scanners provide the opportunities to reduce the total time required to obtain both types of images and enable simultaneous correlation of high-resolution structural images with functional biochemical processes [13]. By far, ^18^F-fludeoxyglucose (^18^F-FDG), a glucose analog, is the most commonly used radioactive isotope for clinical applications. Due to the high background uptake of the normal brain parenchyma and limited specificity in distinguishing between progressive tumor and post-treatment inflammation [14], ^18^F-FDG PET is not routinely used for imaging brain tumors. However, ^18^F-FDG PET provides diagnostic information in the assessment for cognitive impairment and dementia [15, 16] and plays a critical role in guiding surgical resection for patients with epilepsy [17], with distinct hypometabolism seen in most non-lesional (MRI-negative) temporal lobe epilepsy [18, 19]. While the discovery and implementation of new radiotracers provide value in evaluating various tumor and neurologic diseases, the uptake only reflects the accumulation of radioactive substances.

**Fig. 1:**
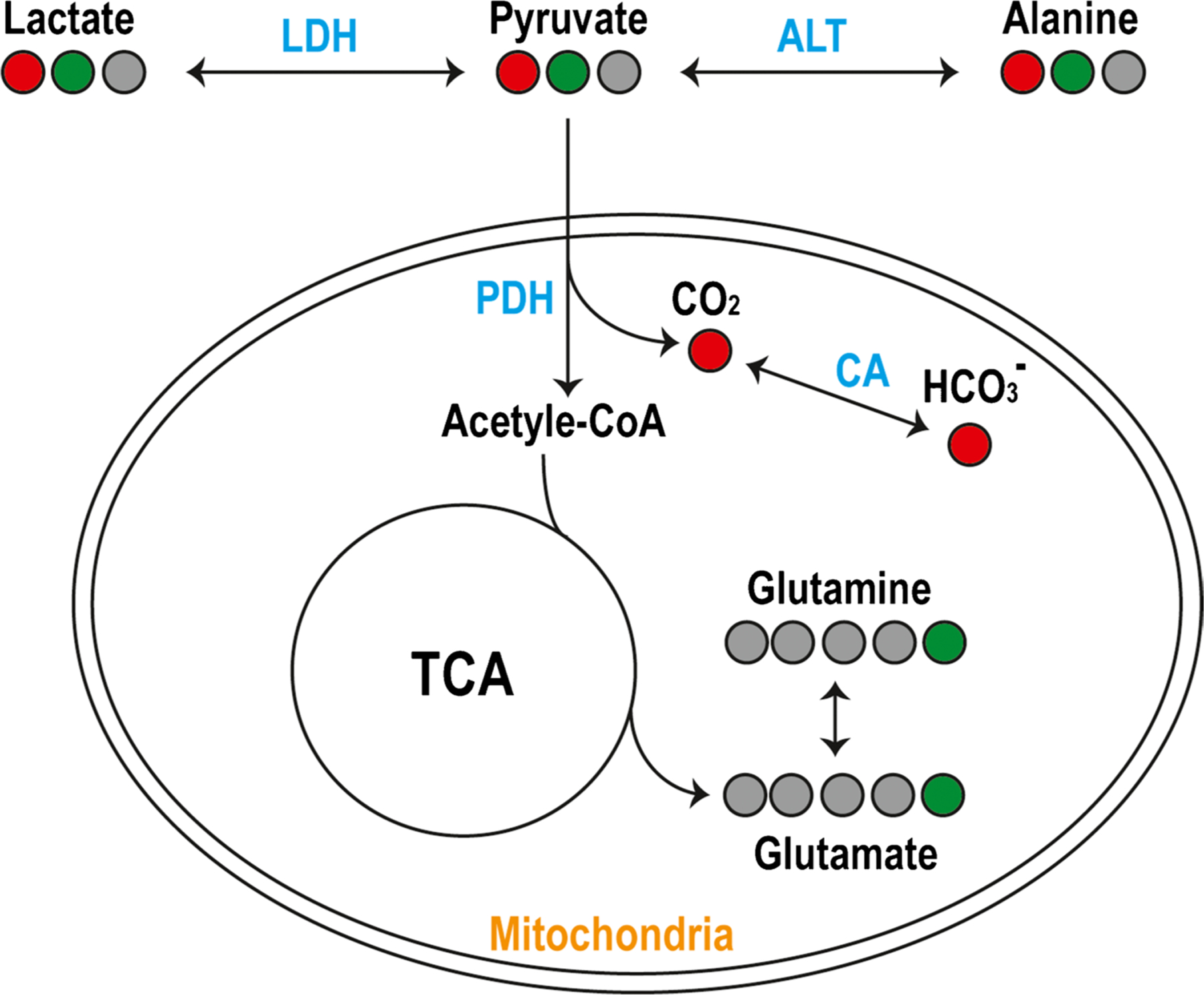
Diagram of observable metabolic pathways using hyperpolarized [1-^13^C]pyruvate (red) and [2-^13^C]pyruvate (green) in the brain metabolism. [1-^13^C] converts to [1-^13^C]lactate via lactate dehydrogenase (LDH), [1-^13^C]alanine via alanine transaminase (ALT) in subcutaneous tissue and muscle outside the brain [43], and ^13^C carbon dioxide (^13^CO_2_) via pyruvate dehydrogenase (PDH) within the mitochondria, which is then catalyzed to ^13^C-bicarbonate by carbonic anhydrase (CA). [2-^13^C]pyruvate provides direct measurements of [2-^13^C]lactate, [5-^13^C]glutamate, and other TCA intermediates

HP ^13^C MR metabolic imaging is a new molecular imaging modality that provides information about real-time metabolism [20–22]. Several reviews have described the method in detail [23, 24]. Fundamentally, this technique pre-polarizes ^13^C-enriched naturally occurring molecules (stable isotopes) using dissolution dynamic nuclear polarization (d-DNP) developed in 2003 [20] by transferring polarization of free radicals or electronic paramagnetic agent to ^13^C-labeled probes, delivers them intravenously, and measures the dynamic changes in ^13^C signals using MRS or frequency selective imaging acquisitions. [1-^13^C]pyruvate, an endogenous metabolite, is the most widely studied HP ^13^C probe. It converts to [1-^13^C]lactate via lactate dehydrogenase (LDH), [1-^13^C]alanine via alanine transaminase (ALT), and ^13^C carbon dioxide (^13^CO_2_) via pyruvate dehydrogenase (PDH) within the mitochondria, which is then catalyzed to ^13^C-bicarbonate by carbonic anhydrase (CA) (Fig. 1) [25]. This highlights [1-^13^C]pyruvate’s unique position to probe anaerobic glycolysis and mitochondrial oxidative metabolism. HP [1-^13^C]pyruvate has been applied in preclinical models and has shown promising results for monitoring brain tumor growth and assessing response to therapy [26–31], assessing aerobic metabolic abnormality and monitoring treatment in traumatic brain injury (TBI) [32, 33], and detecting neuroinflammation in multiple sclerosis [34]. However, the enhanced signal decays as polarization returns to the natural equilibrium after 1–2 min. Similar to the half-life in PET, the T1 relaxation time of the probes determines how long the signal lasts, which in turn, requires fast acquisitions to obtain the dynamic conversion before the signal decays away. The T1 relaxation times of commonly used HP ^13^C probes were summarized in Wang et al. [24]. Of these probes, [1-^13^C]pyruvate has a relatively long T1 relaxation time, ~ 60 s. Additionally, due to the nature of hyperpolarization, the signal can only be used once, creating a need for creative acquisition schemes to fully utilize the available signal.

PET radiolabeled tracers have high sensitivity, but their utility for metabolic imaging is limited by the absence of chemical information—that is, PET can detect tracer uptake but cannot resolve metabolic conversion. The added concerns related to radiation dose from the injected tracer, particularly for studies in healthy subjects, further limit its utilization in neuroscience studies. ^1^H MRS provides chemical information by directly resolving metabolites, providing insight into the brain’s steady-state metabolism. However, it is limited by the low concentration of endogenous metabolites [35], resulting in coarse resolution and long acquisition times that acquire a steady-state snapshot of metabolism that is not fully representative of dynamic processes [36]. HP ^13^C MR metabolic imaging provides dynamic information of metabolic processes and complements both PET and ^1^H MRS’s major weaknesses, by obtaining metabolic dynamic conversions [37] on a rapid timescale.

## Considerations in translating dynamic hyperpolarized ^13^C metabolic imaging to clinical brain studies

While cellular and preclinical HP ^13^C studies have been used to inform and aid in the design of clinical translation, their results are impacted by the use of anesthesia, which is known to alter hemodynamics, metabolism, and functional connectivity [38, 39], and other physiological and evolutionary differences. Additionally, for brain studies, the blood–brain barrier (BBB), an anatomic and physiologic barrier that protects the brain by tightly regulating the passage of molecules, is an important factor that needs to be considered. Small molecules, such as glucose and amino acids, can cross the cell membrane through diffusion and transporters. Pyruvate transport and uptake across the BBB in anesthetized animals have been shown to be rate-limiting [40]. These questions can only be addressed in human brain studies, which further encourage clinical translation. The first human HP [1-^13^C]pyruvate study was reported at the University of California San Francisco in 2013 on a cohort of patients with prostate cancer [41]. The first human brain studies published in 2018 using [1-^13^C]pyruvate have established the safety and feasibility of the imaging agent in patients with brain tumors [42, 43]. The first study [41] was accomplished using a prototype polarizer in a cleanroom to provide the sterile solution for human use, while new clinical polarizers, SPINlab (GE Healthcare), are now commercially available and have been installed in more than 20 academic medical centers worldwide for human studies.

**Fig. 2:**
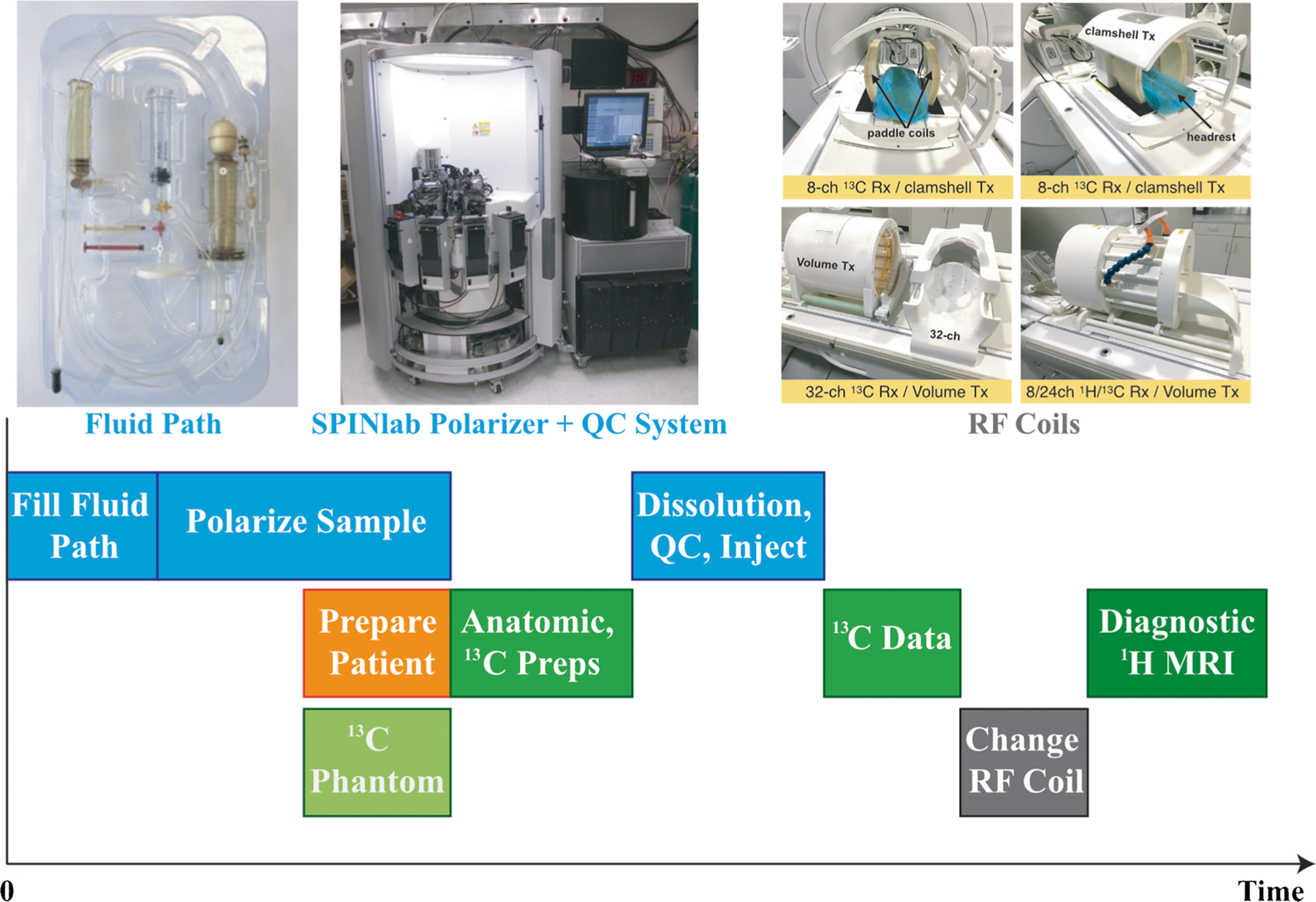
Schematic describing the logistics associated with preparing the ^13^C sample, scanner, and patient. The example of RF coils was from Autry et al. [46]

Previous review articles have summarized key factors required for clinically translating this novel imaging method [24, 44, 45]. The time sensitivity and one-use magnetization associated with HP experiments mean that the development of acquisition procedures, radio-frequency (RF) hardware design, and sequences used to acquire HP ^13^C data are critical. The procedures include the preparation of the pyruvate using a pharmacy kit (containing a sterile fluid path (Fig. 2) and reagents) used for preparing sterile ^13^C probes, hyperpolarization, and quality control (QC) before the injection. The steps for QC [46], including sterilizing the polarizer components and verifying the sample pH, temperature, volume, concentration, and polarization, have been optimized to decrease the time to injection from 88 (74 114) (mean (min max)) [43] to 58 (49 83) s [46] in patients with glioma. Several ^13^C RF multichannel coils have been developed to obtain ^13^C signals with distinct advantages [47, 48]. The choice of use could depend on the regions of interest, head size, and the acquisition methods (e.g., parallel imaging). However, they are single-tuned and require two separate examinations to acquire diagnostic-quality ^1^H MRI data, extending the total time that the patient needs to be in the scanner and inefficiently allocating resources. A dual-tuned ^13^C/^1^H coil was recently used in an integrated ^13^C/^1^H examination [46] and demonstrated decent data quality and improved patient comfort. Compared to the preclinical studies that use small fields of view (FOVs), dynamic HP ^13^C imaging with volumetric coverage and imaging of human brains require far greater FOV coverage. A number of pulse sequences have been developed, such as echo planar spectroscopic imaging (EPSI) [43, 49], frequency-specific echo planar imaging (EPI) [50–53], and IDEAL (iterative decomposition with echo asymmetry and least squares estimation) [54, 55], and discussed in a recent review article [56]. The dynamic data are then reconstructed [45, 55, 57], combined [58] (when acquired with multichannel RF coils), and summed or fitted with a kinetic model to estimate the rates of conversion, for example, from pyruvate to lactate (*k*_PL_) and from pyruvate to bicarbonate (*k*_PB_) for [1-^13^C]pyruvate [55, 59].

HP ^13^C probes that have been used for human brain are [1-^13^C]pyruvate [43, 46, 47, 55, 60, 61] and [2-^13^C]pyruvate [62], with the carbon in the C1 or C2 position labeled with ^13^C. From these studies, the suggested logistics for the preparation of the imaging agent and data acquisition for patient studies are represented in Fig. 2. The fluid path is filled with pyruvate by a licensed pharmacist and placed in the SPINlab system to be polarized for approximately 2 h. Concurrently, the patient will be prepared, their vital signs evaluated, and an intravenous line inserted; while the scanner, RF coil, and pulse sequence to be verified with a phantom scan. Once the polarization and phantom scan have been completed, the patient will be positioned in the scanner and reference anatomic ^1^H images obtained using the body coil or dual-tuned ^13^C/^1^H coil. Once the pre-exam checklist is completed, the sample dissolution will be initiated, QC performed, and the resulting solution captured in the syringe. The syringe will be rapidly transported into the scan room; with the pharmacist signaling approval, the ^13^C tagged compound will be injected into the patient using an automatic injector. Acquisition of the ^13^C data will begin at time of injection or shortly after the completion of saline flush. The remaining ^1^H imaging sequences for clinical use will be obtained to complete the examination using the dual-tuned ^13^C/^1^H coil or after switching to a standard ^1^H coil.

## Emerging human brain applications of dynamic hyperpolarized ^13^C metabolic imaging

For brain studies, HP [1-^13^C]pyruvate has been applied in healthy controls [55, 60]; patients with glioma [42, 43, 46], brain metastasis [42, 63], and TBI [64]; and pediatric patients with CNS tumors [65]. In the following section, we will discuss these most recent clinical applications.

Understanding metabolism in the normal brain is essential to improving our understanding of the biological processes in the diseased brain. The recent healthy volunteer studies exhibited higher [1-^13^C]pyruvate, [1-^13^C]lactate, and ^13^C-bicarbonate in the gray matter compared to white matter [55, 60] and regional variations across the subjects [60]. Establishing the reproducibility of data acquisition, processing, and analysis methods is especially important for providing results that are used for the clinical management of patients. Two volunteers received repeated acquisitions varying from 30 min to 107 days and demonstrated consistency in *k*_PL_ and *k*_PB_ values within the white matter [46]. More recently, a study reported a significant decrease in lactate production associated with age from 28 healthy human subjects [61], providing similar results of the reduction in nonoxidative metabolism with aging in studies using PET agents [66]. This suggests a new application for HP ^13^C metabolic imaging to investigate age related metabolic changes in the brain.

**Fig. 3:**
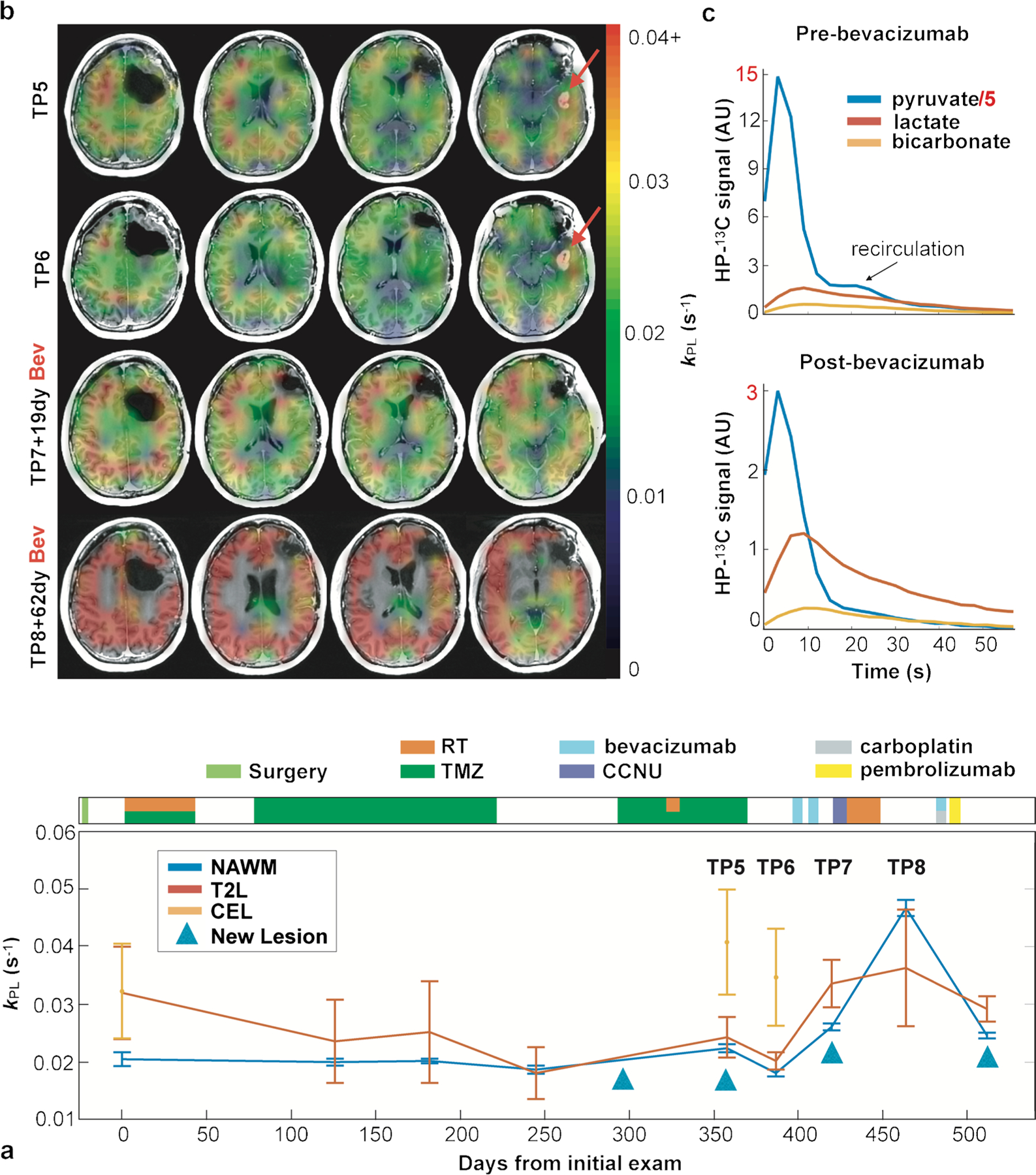
Serial *k*_PL_ within normal-appearing white matter and anatomic lesions from a patient with IDH mutant glioblastoma over 9 scans spanning 512 days (**a**). *k*_PL_ increased in the lesion when new lesions developed. During time points TP5–TP8, the emergence of a new gadolinium-enhancing lesion with elevated *k*_PL_ (red arrows) disappeared following treatment with bevacizumab and subsequent global elevation of *k*_PL_ (**b**). The dynamic data showed that overall lower ^13^C signals after treating with bevacizumab, but the conversion rate to [1-^13^C]lactate was increased (**c**). Figure adapted from Autry et al. [46]

The fundamental basis of using [1-^13^C]lactate in brain tumors relies on the well-known Warburg effect, excessive production of lactate (aerobic glycolysis) in tumor cells [67]. The initial studies in brain tumors were acquired using single-slice dynamic EPSI acquisition [42, 43]. The results in patients with stable disease showed that the ratios of ^13^C-bicarbonate to [1-^13^C]pyruvate (bicarbonate/pyruvate) and [1-^13^C]lactate to [1-^13^C]pyruvate (lactate/pyruvate) were lower in the T2 lesion than those in the normal-appearing brain, with low levels of bicarbonate/pyruvate and elevated lactate/pyruvate ratio in progressive disease [43]. With the development of improved acquisition methods, e.g., multi-slice dynamic EPI acquisition [51], it is possible to longitudinally track the metabolic changes in the regions of interest. Autry et al. [46] reported serial HP ^13^C MR examinations in patients with recurrent glioma. The conversion rates (*k*_PL_, *k*_PB_) within the normal-appearing white matter in the patients who received standard of care treatment were similar to those in healthy controls. Patients with tumor progression showed relatively higher conversion rates to [1-^13^C]lactate (*k*_PL_) in the contrast-enhancing and non-enhancing lesions relative to those in the normal-appearing white matter. In a patient who had been followed for 512 days with 9 HP ^13^C examinations, *k*_PL_ increased in the lesion when new lesions developed (Fig. 3). These encouraging results demonstrate the utility of HP ^13^C imaging for evaluating patients with glioma.

**Fig. 4:**
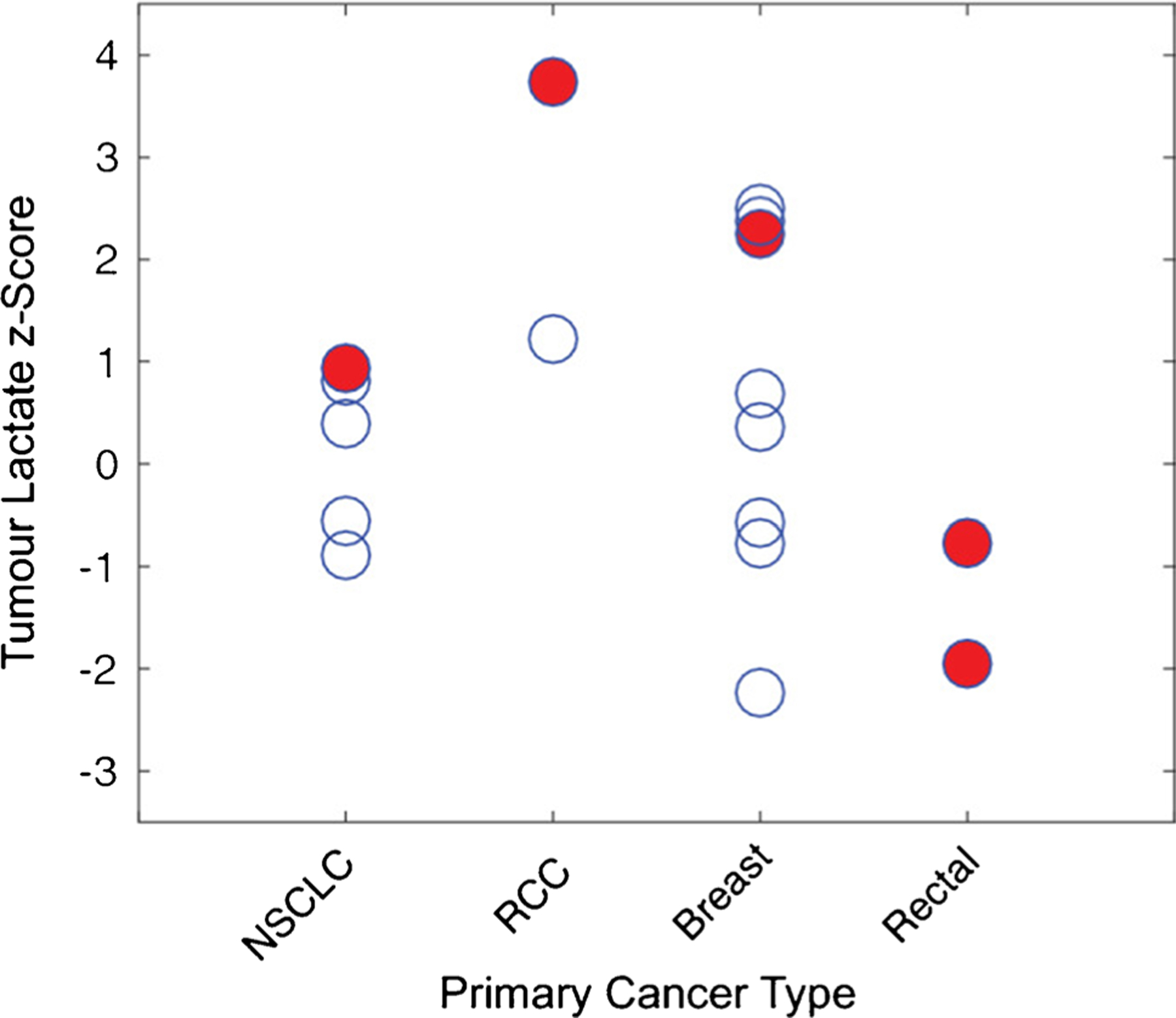
Lactate *z*-scores of new lesions from 11 patients with intracranial metastases. The red circles represent the lesions that progressed at 6 months of post-treatment follow-up or at the time of death, while these open circles are stable or responding lesions. Figure adapted from Lee et al. [63]

Other [1-^13^C]pyruvate HP ^13^C MR metabolic brain imaging studies also include (1) safety and feasibility study in pediatric patients with CNS tumors (diffuse intrinsic pontine glioma, DIPG; craniopharyngioma; medulloblastoma) [65]. This study demonstrated preliminary safety profile in 6 patients at dosages of 0.34 and 0.43 mL/kg. The latter is an adult dosage that was determined in an initial clinical trial [41]. (2) Initial study in patients with brain metastasis found varied [1-^13^C]lactate production in the lesion [42]. Metabolic heterogeneity in brain metastasis was confirmed in a recent study, and progression was associated with the highest lactate *z*-scores, converted from the mean and standard deviation of ^13^C lactate signals for each region [63] (Fig. 4). (3) HP [1-^13^C]pyruvate MR metabolic imaging has been applied to two patients with mild acute TBI [64]. One patient had increased [1-^13^C]lactate over the total ^13^C-labeled metabolite signals (TC) at the injury site, and both patients had decreased ^13^C-bicarbonate over TC in the injured hemisphere. This is the first study demonstrating the feasibility of using dynamic HP [1-^13^C]pyruvate to detect altered downstream glucose metabolism and mitochondrial dysfunction in TBI.

While ^13^C-bicarbonate converted from [1-^13^C]pyruvate has been used as a surrogate biomarker for mitochondria oxidative metabolism, it is nonspecific. Its signal intensity is altered by changes in pH catalyzed by CA [68, 69]. Alternatively, [2-^13^C]pyruvate can be incorporated into the TCA cycle (tricarboxylic acid cycle) intermediates rather than being released as ^13^CO_2_, providing direct measurements of [5-^13^C]glutamate, and other TCA intermediates (Fig. 1). Thus, [2-^13^C]pyruvate allows simultaneous measurement of glycolysis through its conversion to [2-^13^C]lactate and oxidative metabolism to [5-^13^C]glutamate, making it an attractive substrate for evaluating cancer and neurologic diseases. However, there are technical challenges in obtaining these metabolites over large spectral bandwidth in a short acquisition window (short T1, ~ 40 s). Initial HP [2-^13^C]pyruvate studies were performed on healthy volunteers using non-localized dynamic 1D spectroscopy [62]. Sufficient polarization was achieved to observe the TCA cycle metabolites [5-^13^C]glutamate and [1-^13^C]citrate, along with [2-^13^C]lactate. Whole-brain *k*_PL_ values derived from [2-^13^C]pyruvate were consistent with those from [1-^13^C]pyruvate. In a preliminary study with two patients with IDH mutant glioma using 2D chemical shift imaging (non-dynamic), a reduction in the ratio of [5-^13^C]glutamate/[2-^13^C]pyruvate was seen in the T2 lesion compared to the contralateral normal brain, which is consistent with known metabolic reprogramming in IDH mutation [70]. Volumetric, dynamic HP [2-^13^C]pyruvate EPI acquisitions were recently developed and applied to healthy volunteers and patients with glioma [71], which provides the feasibility of using this probe to assess response to therapy for patients with glioma [72–74].

A few other probes have been applied in preclinical models but have not been translated for clinical applications. These candidates in tumors [24, 75] and neurologic diseases [76, 77] have been discussed previously. Similar to PET probes, obtaining US Food and Drug Administration (FDA) regulatory approval is required before starting human studies.

## Challenges in technology of hyperpolarized ^13^C metabolic imaging

With the promising metabolic assessments enabled by HP ^13^C MR metabolic imaging, it is increasingly being used in various human organ systems. These advancements are hindered by the array of required specialized resources, including the pharmacy resource, SPINlab polarizer, ^13^C RF coils, and a team of engineers and scientists, that are mandatory to perform such study.

### Hardware

Currently, the SPINlab instrument can simultaneously polarize up to four samples with regeneration after 12 samples per day. This limits the number of patients that can be performed. With each HP ^13^C injection requiring a sterile fluid path, the total cost is comparable to that of PET study despite short acquisition time. There are ongoing efforts (academically and by GE Healthcare) to reduce the cost and allowing polarizing more samples are needed for further clinical translation. Additionally, as previously described, the choice of receive coil to detect ^13^C signals will impact the quality of data as well as patient handling.

### Acquisition

**Fig. 5:**
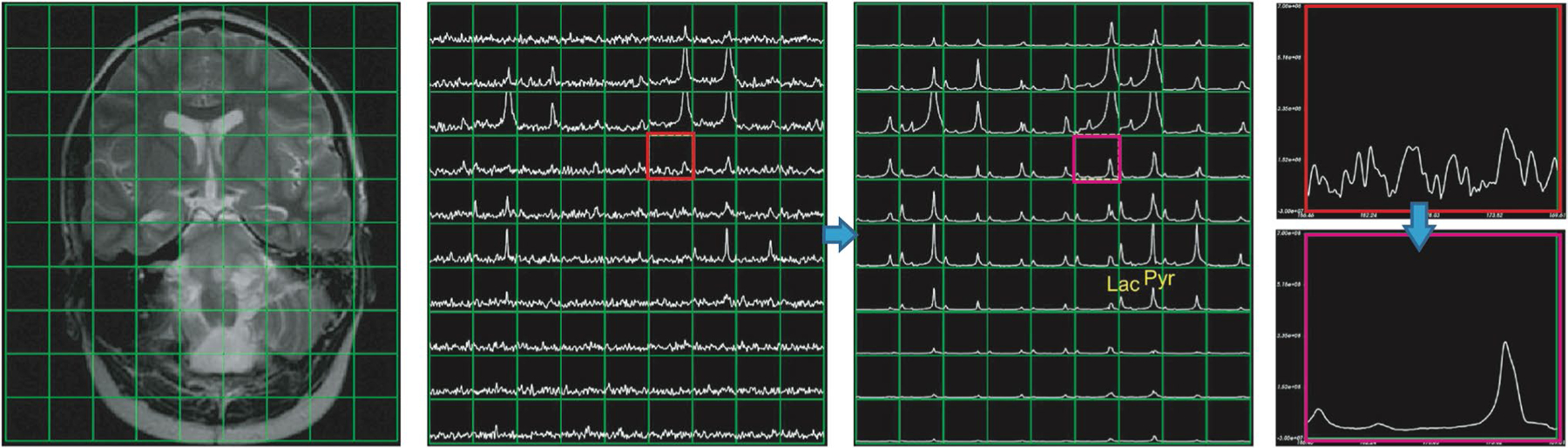
The spectra at 15 s from the start of data acquisition using 2D dynamic EPSI from a 12-year-old patient with DIPG. Whitened singular value decomposition channel sum and tensor rank truncation-image enhancement significantly improved the SNR. Figure adapted from Chen et al. [79]

Although remarkable development has been achieved on acquisition methods, majority of brain HP ^13^C-pyruvate data are acquired at the spatial resolution of 1.5 × 1.5 × 1.5 cm^3^ with 3 s temporal resolution, which is not sufficient to capture spatial heterogeneity for all brain studies. Improvements to reduce the time to injection with optimized sample preparation, use long T1 probes, or further optimize fast metabolic imaging methods [56] will provide better signal-to-time ratio (SNR) to improve spatial resolution. A novel multi-resolution EPI sequence with an in-plane resolution of 7.5 × 7.5 mm for pyruvate and 15 × 15 mm for lactate has recently been developed and demonstrated significantly reduced signal bleeding in from the periphery [52]. This is particularly for the regions around the superior sagittal sinus and white matter with high pyruvate signals from highly perfused regions [78]. For further improvements in SNR, image denoising approaches have recently been shown to increase the sensitivity of HP ^13^C studies. Techniques based on higher-order singular value decomposition (SVD), a generation of multidimensional matrix SVD, have significantly improved SNR for [1-^13^C]lactate and ^13^C-bicarbonate [79, 80]. Figure 5 illustrates an example using tensor rank truncation-image enhancement, which applies higher-order SVD followed by a low-rank approximation. With innovative acquisition and post-processing methods, it is possible to achieve the spatial resolution that is similar to PET images or fMRI. It is worth noting that many of the institutions use homegrown acquisition schemes and processing methods. To conduct large-scale studies, on the scale of the Human Connectome Project, further standardization will be needed to ensure consistency and interpretation of the metabolic data.

### Physiology

An important factor that decides the signal quality is the tracer delivery—flow, perfusion, and active transport. The quantity delivered will directly impact the amount of measured tracer compound and converted metabolites. For example, DIPG is a tumor located in the weakly perfused brain stem. Higher ratios of [1-^13^C]lactate to [1-^13^C]pyruvate were seen in the lesion while [1-^13^C]pyruvate signal was relatively low [65]. In another case shown in Fig. 3, the patient had the enhancing lesion resolved after receiving the anti-angiogenic agent bevacizumab, along with the global increase of *k*_PL_ within the normal-appearing white matter [46]. The dynamic data showed that overall lower ^13^C signals after treating with bevacizumab, but the conversion rate to [1-^13^C]lactate was increased. These two cases suggested that reduced pyruvate delivery either from low perfusion or reduced vascular permeability causes rapid conversion to its downstream metabolites. Since [1-^13^C]pyruvate enters the blood–brain barrier (BBB) via a monocarboxylate transporter, this effect could be due to increased transportation. The role of MCT has been found to be critical for detecting HP [1-^13^C]pyruvate-to-[1-^13^C]lactate [81]. Both MCT1 and MCT4 were significantly increased in IDH mutant glioma and glioblastoma [82, 83]. Besides, the conversion measured also depends on several factors such as NADH levels and LDH activity [25].

## Conclusion and perspectives

This review has highlighted the promising technology of HP ^13^C MRI to acquire dynamic metabolic information, which are complementary to existing anatomic and functional imaging methods. As a technology undergoing rapid development, there are significant hardware, software, and physiological related challenges. Resolving these challenges will be key to wider adoption for clinical diagnostics and measuring early treatment response as well as biological studies.
